# Possible Long-Term Effects of Childhood Maltreatment on Cognitive Function in Adult Women With Posttraumatic Stress Disorder

**DOI:** 10.3389/fpsyt.2020.00344

**Published:** 2020-04-24

**Authors:** Michi Nakayama, Hiroaki Hori, Mariko Itoh, Mingming Lin, Madoka Niwa, Keiko Ino, Risa Imai, Sei Ogawa, Atsushi Sekiguchi, Mie Matsui, Hiroshi Kunugi, Yoshiharu Kim

**Affiliations:** ^1^ Department of Behavioral Medicine, National Institute of Mental Health, National Center of Neurology and Psychiatry, Tokyo, Japan; ^2^ Department of Psychiatry and Cognitive-Behavioral Medicine, Nagoya City University Graduate School of Medical Sciences, Nagoya, Japan; ^3^ Graduate School of Humanities and Social Sciences, Nagoya City University, Nagoya, Japan; ^4^ Department of Clinical Cognitive Neuroscience, Institute of Liberal Arts and Science, Kanazawa University, Kanazawa, Japan; ^5^ Department of Mental Disorder Research, National Institute of Neuroscience, National Center of Neurology and Psychiatry, Tokyo, Japan

**Keywords:** posttraumatic stress disorder, childhood maltreatment, sexual abuse, cognitive function, women

## Abstract

Accumulated evidence shows that individuals with posttraumatic stress disorder (PTSD) have compromised cognitive function. PTSD is associated with childhood maltreatment, which also can negatively affect cognitive function. It is therefore possible that cognitive dysfunction in adult patients with PTSD can be due at least partly to childhood maltreatment, although little is documented on this issue. Here we aimed to examine the possible effect of childhood maltreatment on cognitive function in adult patients with PTSD. A total of 50 women with DSM-IV PTSD and 94 healthy control women were enrolled. Most of the patients developed PTSD after experiencing interpersonal violence during adulthood. History of childhood maltreatment was assessed using the Childhood Trauma Questionnaire (CTQ). Cognitive functions were assessed by the Repeatable Battery for the Assessment of Neuropsychological Status (RBANS). Compared to controls, patients reported significantly more experiences of all types of childhood maltreatment as assessed by the CTQ and showed significantly poorer performance on immediate memory, language, attention, and the total score of RBANS. In patients, sexual abuse scores were significantly negatively correlated with RBANS language (*p* < 0.001) and total score (*p* = 0.005). Further analyses revealed that PTSD patients with childhood sexual abuse had even poorer cognitive function than those without the abuse. In controls, no significant correlation was found between CTQ and RBANS scores. These results suggest that childhood maltreatment, specifically sexual abuse, may lead to persistent cognitive impairment in individuals with PTSD. Our findings might underscore the importance of early detection and intervention of childhood maltreatment, which will be achieved by careful observation of, and listening to, maltreated children in education and welfare scenes as well as clinical settings.

## Introduction

Posttraumatic stress disorder (PTSD) is a debilitating psychiatric condition that can develop after a major traumatic event, often leading to a chronic course and severe functional impairment. Lifetime prevalence of PTSD is estimated at approximately 3.9% worldwide (1). This disorder is characterized by intrusion symptoms associated with the traumatic event, avoidance, hyperarousal, and negative alterations in cognitions and mood (2).

Accumulated evidence shows that PTSD is associated with compromised cognitive functions in a range of domains, including verbal memory, working memory, attention, executive function, and language (3, 4). In line with this, we have recently reported significantly lower performance in wide-ranging cognitive domains, including immediate memory, visuospatial construction, language, attention, delayed memory, and global cognitive functioning, in female patients with PTSD compared to control women (5). However, it remains unclear whether such cognitive dysfunction in PTSD is caused by the illness itself, or by some other factors (e.g., childhood adversity) that relate to risk of this disorder, or by both. Cognitive impairment in individuals with PTSD is of clinical importance as it can predict low social functioning (6) and poor response to cognitive behavioral therapy (7). Moreover, individuals with PTSD are shown to be at higher risk of developing dementia (8). While the mechanisms by which PTSD confers risk for dementia are not well understood, one potential mechanism may be associated with the (traumatic) stress. It is well documented that chronic or excessive stress causes hypothalamic-pituitary-adrenal (HPA) axis dysfunction and increased inflammation, both of which can have detrimental effects on the brain including the hippocampus, thereby possibly leading to dementia.

Childhood maltreatment, which encompasses experiences of emotional abuse/neglect, physical abuse/neglect, and sexual abuse, has repeatedly been reported to increase risk of developing PTSD when exposed to a traumatic event in later life (9, 10). Childhood maltreatment has also been associated with impaired cognitive function (11, 12). It is thus possible that the cognitive impairments in adults with PTSD are caused not only by the disorder itself but by the lasting effect of childhood maltreatment. Indeed, a meta-analytic study targeting children has demonstrated that those with familial trauma (i.e., childhood maltreatment), even without PTSD, show significantly lower overall cognitive function compared to healthy control children without such trauma (13). However, little is known about the possible persistent effect of childhood maltreatment on later life cognitive function among adult patients with PTSD.

One approach to disentangling the effect of childhood maltreatment from that of PTSD is to compare cognitive function of non-PTSD individuals having maltreatment history with that of PTSD patients and that of control subjects without maltreatment history; this comparison will uncover nonspecific effects of childhood maltreatment on cognition. At the same time, it would also be necessary to examine effects of maltreatment on cognition *within* a sample of PTSD patients because the maltreatment history may differentially affect cognition between vulnerable individuals like PTSD patients and resilient (healthy) individuals.

There are studies that have examined associations between specific types of childhood maltreatment and different cognitive domains. In patients with schizophrenia spectrum- or bipolar disorders, childhood physical abuse, sexual abuse and physical neglect were significantly associated with poorer working memory, executive function, and general cognitive function (14). In children and adolescents, sexual abuse was negatively correlated with language and memory functions after controlling for other maltreatment types (11). In addition, both PTSD patients (15, 16) and individuals with histories of childhood maltreatment (17, 18) are shown to have smaller hippocampal volumes, suggesting that memory functions subserved by the hippocampus, among various cognitive domains, may be particularly affected in PTSD patients with childhood maltreatment history.

This study aimed to investigate the effect of childhood maltreatment on cognitive function in adult patients with PTSD and in healthy controls. We only included female subjects, as this study built on our previous findings of cognitive dysfunction in civilian women with PTSD (5). History of childhood maltreatment was retrospectively ascertained by an established self-report questionnaire, and cognitive function was measured by a standardized neuropsychological test battery. We first examined correlations between childhood maltreatment and cognitive function within each diagnostic group. Then, cognitive function was directly compared between PTSD patients with maltreatment, those without maltreatment, controls with maltreatment, and those without maltreatment, which were classified by a well-defined cutoff score of the questionnaire. Our primary hypothesis was that PTSD patients with childhood maltreatment would show the greatest cognitive impairment, given that the combination of maltreatment and PTSD, both of which can negatively affect cognition, would yield even greater cognitive deficits compared to either of them alone. We also hypothesized that memory functions would be particularly impaired in the PTSD patients with childhood maltreatment history.

## Methods

### Participants

Details of participant recruitment have been described previously (5). Initially, 61 civilian female patients with PTSD and 96 non-trauma-exposed healthy control women were enrolled. Of these subjects, 11 patients and 2 controls were excluded based on their low RBANS total scores (as detailed below). Consequently, 50 patients with PTSD and 94 healthy controls were included in all analyses in this study. All participants were native Japanese speakers residing in metropolitan areas in Japan. They had no severe physical illness or apparent intellectual disability. All patients had already been diagnosed as having PTSD by their attending clinicians. The experience of traumatic events and diagnosis of PTSD were confirmed by the validated Japanese version (19) of the Posttraumatic Diagnostic Scale [PDS; (20)]. The PDS was also administered to healthy controls in order to evaluate the presence/absence of traumatic experiences and, if present, excluded from this study. Additionally, the Mini International Neuropsychiatric Interview (21) was administered to identify any other Axis-I disorders as well as PTSD in patients and to ascertain the absence of any Axis-I disorders in controls.

This study was approved by the ethics committees of the institutes involved, and was conducted in accordance with the Declaration of Helsinki. Written informed consent was obtained from all participants after they had received a detailed explanation of the study. A significant subset of the present participants (94 of the total 144 participants: 65.3%) had been included in our previous study on the association between PTSD and cognitive function (5).

### Assessment of Trauma Experience, PTSD Diagnosis, and Symptomatology

The PDS was created in accordance with the diagnostic criteria of PTSD in DSM-IV (20). This scale comprises 4 parts that evaluate traumatic experiences (Parts 1 & 2), PTSD symptoms during the past month (Part 3), and the associated functional impairments (Part 4). In the present study, we administered Parts 1 & 2 to all participants for the assessment of presence/absence of traumatic experiences and Parts 3 & 4 to only patients for the assessment of PTSD diagnosis. We have previously shown that the PTSD diagnosis concordance rate between the PDS and the Clinician-Administered PTSD Scale (22), a structured interview for the diagnosis of PTSD, was very high (95.1%, κ = 0.90) (23).

PTSD severity of the patients was assessed using the validated Japanese version (24) of the Impact of Event Scale-Revised [IES-R; (25)], a 22-item self-report questionnaire measuring the 3 core PTSD symptom clusters: intrusion, avoidance, and hyperarousal. Each item is scored on a 5-point scale of symptom intensity, with higher scores indicating greater symptom severity.

Anxiety symptoms were assessed by the State-Trait Anxiety Inventory [STAI; (26)], a self-report questionnaire widely used to assess anxiety. It consists of 2 subscales for trait (STAI-T) and state (STAI-S) anxiety, both of which comprise 20 items that are scored on a 4-point scale from 1 to 4; higher scores indicate greater anxiety. We used the validated Japanese version (27) of the STAI.

Depressive symptoms were assessed by the Beck Depression Inventory-II [BDI-II; (28)], a 21-item self-report questionnaire widely used to measure depression severity during the past two weeks. Each item is scored on a 4-point scale from 0 to 3, with higher scores indicating more severe depressive symptoms. We used the validated Japanese version (29) of the BDI-II.

### Assessment of Childhood Maltreatment

The Childhood Trauma Questionnaire [CTQ; (30)] was used to assess history of childhood maltreatment. The commonly-used 28-item version of CTQ includes 25 clinical items and 3 validity items. The former 25 items are classified into 5 subscales that assess different types of childhood maltreatment, including emotional abuse, physical abuse, sexual abuse, emotional neglect, and physical neglect. All items are rated on a 5-point scale, with higher scores indicating more severe maltreatment. Cut-off scores for each subscale are defined in the manual of the CTQ (31). The CTQ has demonstrated adequate psychometric properties as indicated by a good fit of the 5-factor structure (30, 32), internal consistency (30, 33), and test-retest reliability (31).

We used the CTQ after translating it from the original English version into Japanese by one of the authors (HH), which was then back-translated into English by another Japanese researcher, and the back-translated English version was sent to and approved by the original author (Professor David Bernstein). Cronbach α coefficients of the 5 CTQ subscales, namely, emotional abuse, physical abuse, sexual abuse, emotional neglect, and physical neglect, in the present sample (n = 144) were 0.93, 0.87, 0.94, 0.91, and 0.72, respectively.

### Cognitive Measurement

Cognitive functioning of participants was measured using the Japanese version (34) of the Repeatable Battery for the Assessment of Neuropsychological Status [RBANS; (35)], a well-established neuropsychological test battery. With 12 subtests, the RBANS can assess immediate memory, visuospatial construction, language, attention, and delayed memory, as well as the total score. Age-corrected standardized scores, with a population mean of 100 and standard deviation (SD) of 15, are calculated for each cognitive domain (34, 35). The RBANS has demonstrated good psychometric properties among clinical and nonclinical populations (34, 36–38). Scoring was done in accordance with the manual guidelines (34, 35).

In the present study, participants with the RBANS total score < 70 (i.e., 2 *SD* below the expected population mean) were excluded, considering that cognitive impairment of this magnitude can suggest the presence of intellectual disability and is unlikely to be accounted for by the effect of childhood maltreatment.

### Statistical Analysis

Averages are reported as “means ± SD”, or “median (25–75th percentile)” where appropriate. Categorical variables were compared using the *χ^2^* test. The *t*-test or Mann-Whitney *U* test was used to examine group differences. Correlations were calculated using Pearson's *r* or Spearman's *ρ*. The use of parametric or nonparametric test was determined based on the nature and distribution of the data. Correlations for the CTQ scores were calculated by Spearman's *ρ* since the CTQ data deviated from the normal distribution. A 2-way analysis of covariance (ANCOVA), controlling for potentially confounding variable(s), was used to examine the effect of diagnosis (i.e., PTSD patients vs. healthy controls) and childhood maltreatment (presence vs. absence based on the CTQ cut-off score), along with their interaction effect, on cognitive function. We dichotomized the CTQ data using the cut-off score, instead of using their original scores, in order to include this variable as a fixed factor, rather than as a covariate, in the 2-way ANCOVA. This was because the original CTQ scores markedly deviated from the normal distribution, and therefore this variable was considered not appropriate for the parametric model.

Statistical significance was set at 2-tailed *p* < 0.05 unless otherwise specified. For the 5 subscales of RBANS, the Bonferroni-corrected p values, i.e., *p* < 0.01 (= 0.05/5), were adopted as statistical significance and the threshold of *p* < 0.05 was considered as statistical trend in order to correct for the multiple comparisons; while this correction was not applied to the total score of RBANS. All statistical analyses were performed using the Statistical Package for the Social Sciences version 25.0 (IBM Corp., Tokyo, Japan).

## Results

### Sample Characteristics

As shown in **Table 1**, patients with PTSD and healthy controls did not significantly differ in any of the demographic variables examined, including age, education level, and current smoking status. Compared to controls, patients reported significantly greater symptoms of anxiety (as assessed by the STAI) and depression (BDI-II) and more childhood experiences of maltreatment as measured by the CTQ 5 domains (**Table 1**). Patients showed significantly poorer performance than controls on RBANS immediate memory (*t* = −3.5, *p* < 0.001), language (*t* = −2.8, *p* = 0.005), attention (*t* = −3.7, *p* < 0.001), and the total score (*t* = −4.6, *p* < 0.001).

**Table 1:** Demographic and psychological characteristics in PTSD patients and healthy controls.

Variable	PTSD patients (n = 50)	Healthy controls (n = 94)	Analysis		
			Statistic	d.f.	p
Age, years: mean ± SD	38.7 ± 10.5	35.0 ± 13.0	^b^ *t* = 1.8	120.0	0.07
Education level^a^: median (25−75 percentile)	3.0 (3.0−4.0)	3.0 (3.0−4.0)	Mann-Whitney *U* = 2143.5		0.46
Smoking: yes, n (%)	9 (18.0)	10 (10.6)	χ^2^ = 1.5	1	0.21
STAI-state: mean ± SD	51.2 ± 9.8	36.4 ± 7.8	*t* = 9.8	142	**< 0.001**
STAI-trait: mean ± SD	62.4 ± 9.0	38.6 ± 9.1	*t* = 14.9	142	**< 0.001**
BDI-II: mean ± SD	31.1 ± 13.2	5.4 ± 4.9	^b^ *t* = 13.3	56.4	**< 0.001**
CTQ: median (25−75 percentile)					
Emotional abuse	16.5 (8.0−21.0)	6.0 (5.0−8.0)	Mann-Whitney *U* = 3923.0		**< 0.001**
Physical abuse	7.0 (5.0−12.25)	5.0 (5.0−5.0)	*U* = 3718.0		**< 0.001**
Sexual abuse	5.0 (5.0−9.0)	5.0 (5.0−5.0)	*U* = 3179.0		**< 0.001**
Emotional neglect	19.5 (13.0−23.25)	11.0 (8.0−15.0)	*U* = 3742.5		**< 0.001**
Physical neglect	9.0 (6.75−11.25)	6.0 (5.0−7.0)	*U* = 3626.0		**< 0.001**

PTSD, posttraumatic stress disorder; STAI, State-Trait Anxiety Inventory; BDI-II, Beck Depression Inventory-II; CTQ, Childhood Trauma Questionnaire; d.f., degree of freedom; SD, standard deviation.

a Coded as follows: 1, junior high school graduate; 2, high school graduate; 3, some college graduate/partial university; 4, university graduate; 5, graduate school graduate.

b Assumption of homogeneity of variance was not satisfied.

Bold p values represent significant results.

Concerning clinical variables of PTSD patients, most (38/50: 76.0%) developed the disorder after experiencing interpersonal violence such as domestic and/or sexual violence; of the 38 patients, 12 experienced the index interpersonal trauma before the age of 6 years old, and the remaining 26 experienced it in later life. There were also several patients who developed PTSD after experiencing other types of traumatic events during adulthood, such as motor vehicle accidents and natural disasters. All patients were outpatients at the time of the experiment. Most of the patients (46/50: 92.0%) had suffered from PTSD for more than 6 months (for patients with PTSD, this illness duration was calculated by subtracting the age of index trauma from the present age of the patient). Many of the patients (37/50: 74.0%) had psychiatric comorbidity including major depressive disorder (29/50: 58.0%), bipolar disorder (2/50: 4.0%), anxiety disorders (22/50: 44.0%), obsessive-compulsive disorder (8/50: 16.0%), and substance abuse/misuse (6/50: 12.0%). Most of them were receiving psychotropic medications such as antidepressants (28/50: 56.0%) and anxiolytics (22/50: 44.0%). For PTSD severity, the IES-R intrusion, avoidance, hyperarousal symptom scores and total score were 17.1 ± 8.4, 18.1 ± 8.4, 13.7 ± 5.6 and 48.9 ± 18.0, respectively.

In patients, CTQ physical abuse scores were significantly positively correlated with IES-R intrusion (*ρ* = 0.32, *p* = 0.024) and total score (*ρ* = 0.28, *p* = 0.049), whereas no significant correlations were seen between the other 4 CTQ domains and any of the IES-R 3 clusters or total score (all *p* > 0.05).

### Correlations Between Childhood Maltreatment and Cognitive Function

**Figure 1:**
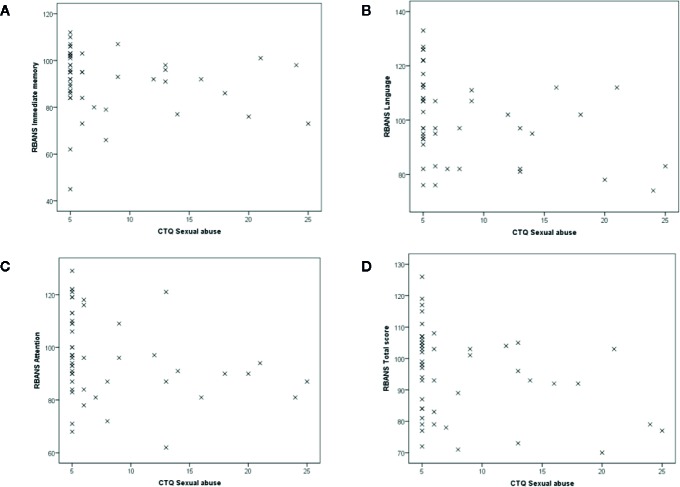
Scatterplot of association between childhood sexual abuse and cognitive functions in patients. Associations of CTQ sexual abuse scores with **(A)** immediate memory, **(B)** language, **(C)** attention, and **(D)** total score of RBANS. CTQ, Childhood Trauma Questionnaire; RBANS, Repeatable Battery for the Assessment of Neuropsychological Status.

Correlations between CTQ scores and RBANS scores are shown in **Table 2**, which were calculated separately for patients and controls. In patients, emotional abuse/neglect and physical abuse/neglect were not significantly correlated with any of the RBANS 5 indices or total score; however, sexual abuse was significantly negatively correlated with RBANS language (*ρ* = −0.45, *p* < 0.001) and total score (*ρ* = −0.39, *p* = 0.005), and was correlated with immediate memory (*ρ* = −0.28, *p* = 0.045) and attention (*ρ* = −0.36, *p* = 0.010) at a trend-level (**Figure 1**).

**Table 2:** Correlations between childhood maltreatment and cognitive function in PTSD patients and healthy controls (calculated by Spearman's ρ).

	PTSD patients (n = 50)						Healthy controls (n = 94)					
	Immediate memory (RBANS)	Visuospatial construction (RBANS)	Language (RBANS)	Attention (RBANS)	Delayed memory (RBANS)	RBANS total score	Immediate memory (RBANS)	Visuospatial construction (RBANS)	Language (RBANS)	Attention (RBANS)	Delayed memory (RBANS)	RBANS total score
Emotional abuse (CTQ)	−0.221	−0.100	0.037	0.012	0.096	−0.039	0.126	−0.010	0.107	0.062	0.194	0.130
Physical abuse (CTQ)	−0.199	0.116	0.061	0.069	0.017	0.016	−0.125	−0.194	−0.038	−0.028	−0.046	−0.142
Sexual abuse (CTQ)	−0.284^†^	−0.054	−0.453^***^	−0.361^†^	0.055	−0.388^**^	0.034	0.052	0.182	−0.010	0.145	0.106
Emotional neglect (CTQ)	−0.125	0.075	−0.113	0.042	0.128	0.002	0.032	0.108	0.113	0.146	0.135	0.156
Physical neglect (CTQ)	−0.231	−0.120	−0.070	−0.033	−0.025	−0.072	0.097	0.096	−0.019	−0.093	0.132	0.067

PTSD, posttraumatic stress disorder; RBANS, Repeatable Battery for the Assessment of Neuropsychological Status; CTQ, Childhood Trauma Questionnaire.

† p < 0.05; ^**^p < 0.01; ^***^p < 0.001.

Correlations were calculated using Spearman's ρ.

In controls, no significant correlation was observed between any of the CTQ domains and RBANS indices.

### Cognitive Function in Subjects With Childhood Sexual Abuse Versus Those Without

To further investigate the relationship of childhood sexual abuse with cognitive function, we subdivided the subjects into those with childhood sexual abuse and those without, and compared cognitive function between these groups. Specifically, using the well-defined cutoff of 5/6 points for the absence/presence of CTQ sexual abuse (31), the subjects were classified into those with childhood sexual abuse and those without. This classification identified 21 patients with childhood sexual abuse and 29 patients without, and also 8 controls with the abuse and 86 controls without; the proportion of individuals with the abuse was significantly higher in patients than in controls [*χ^2^*(1) = 22.8, *p* < 0.001]. Patients with and those without childhood sexual abuse did not significantly differ in age, education level, smoking status, comorbid major depressive disorder, comorbid anxiety disorders, comorbid substance abuse/misuse, use of antidepressants, use of anxiolytics, or PTSD severity as indexed by the IES-R intrusion, avoidance, hyperarousal symptom scores and total score (all *p* > 0.1). In addition, patients with and those without childhood sexual abuse did not significantly differ in trait/state anxiety (as assessed by the STAI) or depressive symptoms (BDI-II) (all *p* > 0.1); while in controls, those with and those without childhood sexual abuse significantly differed in depressive symptoms (BDI-II) (*t* = 3.1, *df* = 92, p = 0.003), but not state/trait anxiety symptoms (both p > 0.1). We therefore decided to control for the BDI-II scores in the following 2-way ANCOVA.

**Figure 2:**
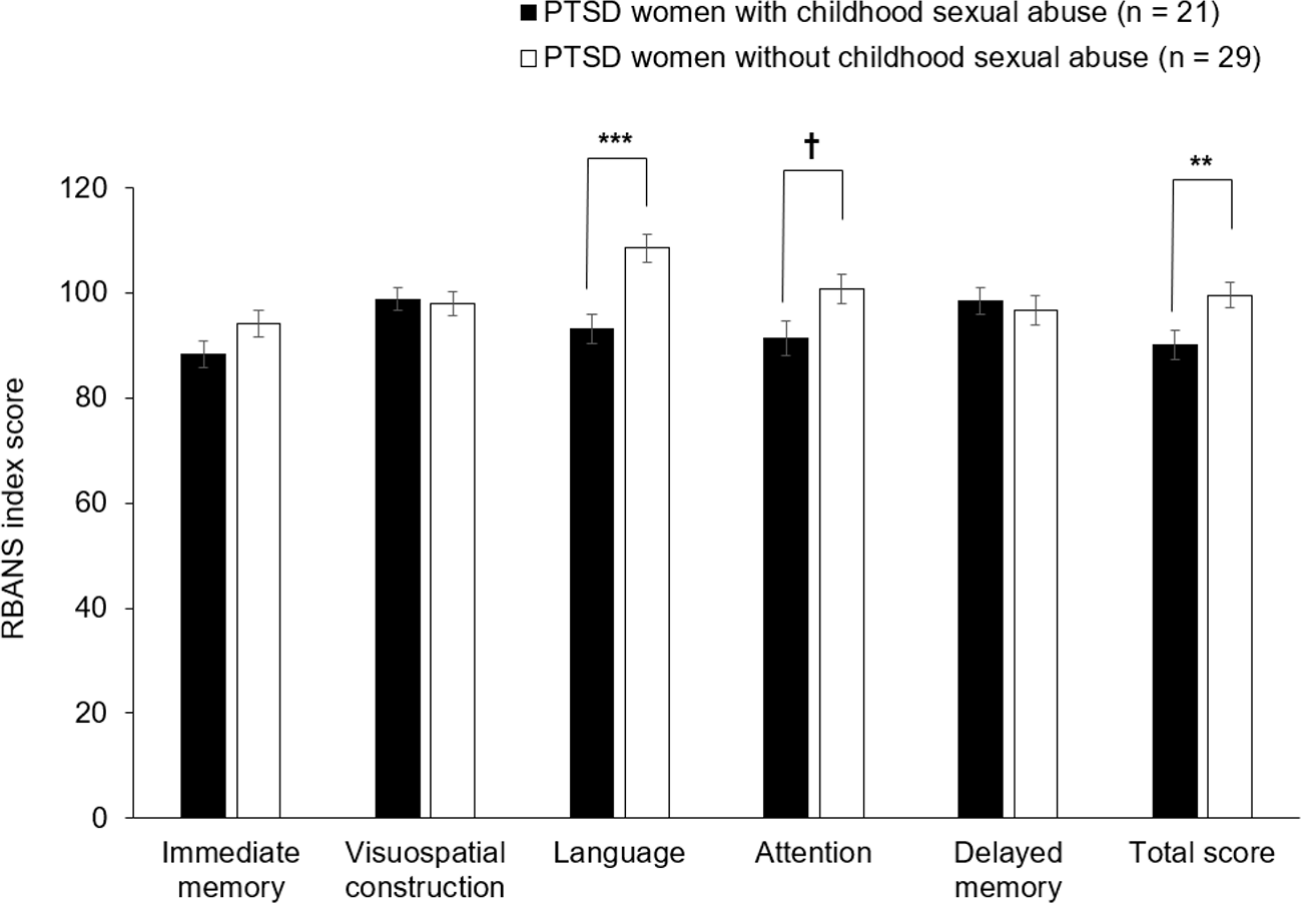
Comparison of the RBANS scores between PTSD patients with and without history of childhood sexual abuse. PTSD patients with history of childhood sexual abuse (n = 21) were defined as those patients whose CTQ sexual abuse scores were 6 or more; while PTSD patients without history of childhood sexual abuse (n = 29) were defined as the remaining patients whose CTQ sexual abuse scores were less than 6. Comparisons were made by the post-hoc pairwise analysis of 2-way analysis of covariance. Error bars indicate SEM. ^†^p = 0.037; ^**^p = 0.011; ^***^p < 0.001. CTQ, Childhood Trauma Questionnaire; RBANS, Repeatable Battery for the Assessment of Neuropsychological Status.

The 2-way ANCOVA examining the effect of diagnosis and childhood sexual abuse (i.e., presence vs. absence) on the RBANS 5 domain and total scores, with the BDI-II scores as a covariate, showed that diagnosis had a significant main effect on the RBANS total score [*F*(1,139) = 5.7, *p* = 0.018] and a trend-level effect on language [*F*(1,139) = 4.1, *p* = 0.044]; that childhood sexual abuse did not have significant main effect on any RBANS indices (all *p* > 0.1); and that diagnosis-by-abuse interaction was significant for language [*F*(1,139) = 11.8, *p* < 0.001] and total score [*F*(1,139) = 7.0, *p* = 0.009]. This analysis further revealed that compared to PTSD patients without childhood sexual abuse, those with the abuse performed significantly more poorly on language (estimated mean difference = 15.2, 95% confidence interval = 7.3 to 23.1, *p* < 0.001) and total score (estimated mean difference = 9.0, 95% confidence interval = 2.1 to 15.9, *p* = 0.011) (**Figure 2**). In addition, **Figure 2** shows that mean scores on these 2 RBANS indices were both around 100 in patients without childhood sexual abuse while they were around 90 in patients with the abuse, suggesting that language and global cognitive function were within normal range in the former patients but compromised in the latter patients.

## Discussion

In this study, we investigated the possible lasting effect of childhood maltreatment on cognitive function in adult patients with PTSD and in healthy controls. We first confirmed significantly lower cognitive performance and more experiences of childhood maltreatment in patients than in controls. Our main finding was that experiences of childhood sexual abuse in patients were associated with poorer cognitive functioning including language and global performance. The subgroup analysis based on the cut-off score showed that patients with sexual abuse had even poorer cognitive function than those without the abuse. The other types of abuse and neglect were not significantly correlated with cognitive functioning in PTSD patients. In controls, no types of abuse or neglect were significantly correlated with cognitive functioning. These results indicate that childhood sexual abuse can have persistent negative impacts on cognitive function specifically in PTSD patients.

Previous studies have examined the effects of childhood maltreatment on later life cognitive function in various populations, such as the general population (12), patients with first-episode psychosis (14, 39), and those with bipolar disorder (39, 40). For example, a systematic review of prospective cohort studies demonstrates that childhood maltreatment has significant negative impacts on later life cognitive functioning (12). In addition, history of childhood maltreatment was associated with poorer cognitive performance in patients with first-episode affective psychosis (14) and in those with bipolar disorder recently recovered from a first manic episode (40). As for PTSD, studies have examined cognitive function in maltreated children with and without this disorder (13, 41), demonstrating cognitive deficits in these children irrespective of PTSD diagnosis (13). However, little has been done to understand the possible long-term effects of childhood maltreatment on cognition in adult patients with this disorder.

Childhood maltreatment is typically repetitive and persistent, and therefore tends to be severe in nature. Moreover, studies have shown that maltreated children become more likely to be bullied by their peers (42) and result in school absenteeism (43). Such environmental deprivation may lead to lower cognitive abilities, especially with respect to crystallized intelligence like language. Our results also show that, of the 5 types of maltreatment, only sexual abuse was associated with poor cognitive functioning. This suggests that the association between maltreatment and cognition can differ depending on maltreatment types, with sexual abuse being specifically associated with impaired cognition. In line with this, a number of studies have reported that childhood sexual abuse is associated with a variety of unfavorable outcomes, including psychosocial problems and psychiatric disorders such as depression, alcohol dependence, eating disorders, and PTSD (44, 45). On the other hand, we found that only physical abuse was significantly associated with more severe PTSD symptoms, particularly intrusion symptoms. These results together suggest that childhood sexual and physical abuse, as compared to the other types of maltreatment, are associated with psychopathology of PTSD. Relatedly, a meta-analysis shows that sexual and physical abuse is related to higher dissociation than are emotional abuse and neglect (46). Nonetheless, it would be worth noting that our results indicated that sexual and physical abuse was associated with *different* aspects of PTSD, i.e., cognitive function and symptom severity (respectively). This differential association may be related to the fact that compared to physical abuse, sexual abuse can be less overt to the surroundings or even to the victim her/himself, as it usually does not leave obvious wounds or physical pain. What makes matters worse is that the awareness of sexual abuse for the victim her/himself requires knowledge regarding gender and sex, which can usually be learned after puberty at least in Japan. Furthermore, sexual abuse may be difficult to verbalize because of social and cognitive barriers compared to physical abuse. For these reasons, there will be a major delay in detection of, and intervention for, sexual abuse, and thus this form of abuse can be long-lasting and consequently may have negative impacts on cognitive function.

An increasing body of evidence shows that childhood maltreatment causes not only psychosocial problems but also alterations in stress-related biological systems including HPA axis dysfunction and increased inflammation. Studies have suggested that childhood maltreatment can have lasting effects on these stress systems (47, 48), at least partly *via* epigenetic mechanisms (49). The HPA axis dysregulation and inflammation, in turn, exert detrimental effects on the brain and negatively affect cognition (50–52). Indeed, neuroimaging studies have demonstrated that childhood maltreatment can cause structural and functional alterations in the brain (18, 50). Furthermore, HPA axis dysfunction (53), increased inflammation (54), and cognitive dysfunction (55) have all been shown to precede the onset of PTSD. These collectively suggest that childhood maltreatment leads to persistent cognitive impairment through the dysregulation of stress response systems, and these psychobiological factors can increase the risk of developing PTSD which in turn will contribute to further cognitive impairment.

In healthy controls, childhood maltreatment was not associated with cognitive impairment. As mentioned earlier, studies have reported impaired cognitive function in maltreated children (56). The non-significant finding in the present study may therefore be attributable to the fact that our control subjects included only a small number of individuals with childhood maltreatment history. Still, the absence of significant association in controls may not be solely ascribed to the type II error; the significant interaction between diagnosis and sexual abuse status suggests that the effects of abuse on cognitive function can be different between patients and controls. Actually, previous studies have not consistently observed the association between maltreatment and worse cognition among the general population, especially in older populations. For example, a large population study of the elderly reported no difference in IQ between the severely abused and non-abused (57). It may be that in older populations the negative effects of childhood maltreatment on cognitive function become less marked and limited, contrary to the apparent effects in children. Considering that the average age of our control subjects was 35.0 years, their psychological/biological resilience may have outweighed any deleterious effects of childhood maltreatment, and such a process might have gradually occurred with advancing age. Taken together, our results suggest the following scenario: childhood maltreatment, specifically sexual abuse, can lead to persistent cognitive impairment in some (but not all) individuals; such childhood abuse and impaired cognition will both confer vulnerability for the development of PTSD when exposed to a traumatic event in later life; and this disorder itself can cause additional cognitive impairment during its onset and illness course.

There were several limitations to the present study. Firstly, the sample size was relatively small, particularly when the subjects were divided into subgroups with and without childhood maltreatment. This may have affected some statistical results. For instance, the number of controls with history of childhood sexual abuse was very small (i.e., n = 8) and therefore the non-significant results may have actually represented type II errors. Secondly, we only included female patients with PTSD. As the observed finding on sexual abuse might be specific to female (and not male) patients with PTSD, future studies that examine the relation between childhood maltreatment, PTSD, and cognitive function in male patients, or both sexes, are warranted. Thirdly, the wide age range of our patients (and controls) may have influenced the results, although the RBNAS is a standardized neuropsychological test that provides age-corrected index scores. For example, the association between childhood maltreatment and adulthood cognitive function may vary with advancing age, such that aging might either mitigate or magnify the effect of maltreatment on cognition during the later life. Fourthly, it would have been better to include another standardized neuropsychological measure, in addition to the RBANS, for more detailed assessment of cognitive function. Finally, we used a retrospective measure to assess childhood maltreatment. According to a meta-analytic study, prospective and retrospective measures of childhood maltreatment show poor agreement (58). Given the assumption that prospective measures are more precise than retrospective measures, our assessment of childhood maltreatment may have biased the results; however, it is often difficult to prospectively ascertain covert forms of maltreatment like sexual abuse during childhood.

In conclusion, we show that PTSD patients with childhood sexual abuse have even poorer cognitive function, including language and global functioning, than those without the abuse. This suggests that while the development of PTSD will cause (additional) cognitive impairment, childhood sexual abuse might have long-term negative effects on cognition in those individuals who later develop this disorder. Our findings may point to the importance of early detection and intervention of childhood maltreatment, which will be achieved by careful observation of, and listening to, maltreated children in education and welfare scenes as well as clinical settings.

## Data Availability Statement

The datasets generated for this study are available on request to the corresponding author.

## Ethics Statement

The studies involving human participants were reviewed and approved by Ethics committee of National Center of Neurology and Psychiatry; Ethics committee of Tokyo Women's Medical University; and Ethics committee of Nagoya City University. The patients/participants provided their written informed consent to participate in this study.

## Author Contributions

MiN and HH designed the study. MiN, HH, MI, ML, MaN, KI, RI, and YK collected the data. MM supervised the conduct of RBANS. HH undertook the statistical analyses. MiN and HH wrote the draft of the manuscript. SO, AS, MM, HK, and YK gave critical comments on the manuscript. All authors contributed to and have approved the final manuscript.

## Funding

This work was supported by Japan Society for the Promotion of Science (JSPS) KAKENHI [19H01047]; Health Labour Sciences Research Grant from the Japanese Ministry of Health, Labour and Welfare [201616028]; grants from the Koyanagi Foundation, the Foundation for Total Health Promotion, and the Mental Health Okamoto Memorial Foundation.

## Conflict of Interest

The authors declare that the research was conducted in the absence of any commercial or financial relationships that could be construed as a potential conflict of interest.

## References

[1] Koenen KC Ratanatharathorn A Ng L McLaughlin KA Bromet EJ Stein DJ Posttraumatic stress disorder in the World Mental Health Surveys. Psychol Med (2017) 47:2260–74. 10.1017/S0033291717000708 PMC6034513 28385165

[2] American Psychiatric Association Diagnostic and Statistical Manual of Mental Disorders (DSM-5). Washington DC: American Psychiatric Publishing (2013).

[3] Johnsen GE Asbjørnsen AE Consistent impaired verbal memory in PTSD:A meta-analysis. J Affect Disord (2008) 111:74–82. 10.1016/j.jad.2008.02.007 18377999

[4] Scott JC Matt GE Wrocklage KM Crnich C Jordan J Southwick SM A quantitative meta-analysis of neurocognitive functioning in posttraumatic stress disorder. Psychol Bull (2015) 141:105–40. 10.1037/a0038039 PMC4293317 25365762

[5] Narita-Ohtaki R Hori H Itoh M Lin M Niwa M Ino K Cognitive function in Japanese women with posttraumatic stress disorder: Association with exercise habits. J Affect Disord (2018) 236:306–12. 10.1016/j.jad.2018.02.061 29482857

[6] Geuze E Vermetten E de Kloet CS Hijman R Westenberg HG Neuropsychological performance is related to current social and occupational functioning in veterans with posttraumatic stress disorder. Depress Anxiety (2009) 26:7–15. 10.1002/da.20476 18800372

[7] Wild J Gur RC Verbal memory and treatment response in post-traumatic stress disorder. Br J Psychiatry (2008) 193:254–5. 10.1192/bjp.bp.107.045922 18757989

[8] Yaffe K Vittinghoff E Lindquist K Barnes D Covinsky KE Neylan T Posttraumatic stress disorder and risk of dementia among US veterans. Arch Gen Psychiatry (2010) 67:608–13. 10.1001/archgenpsychiatry.2010.61 PMC2933793 20530010

[9] McLaughlin KA Koenen KC Bromet EJ Karam EG Liu H Petukhova M Childhood adversities and post-traumatic stress disorder: evidence for stress sensitisation in the World Mental Health Surveys. Br J Psychiatry (2017) 211:280–8. 10.1192/bjp.bp.116.197640 PMC5663970 28935660

[10] Scott KM Smith DR Ellis PM Prospectively ascertained child maltreatment and its association with DSM-IV mental disorders in young adults. Arch Gen Psychiatry (2010) 67:712–9. 10.1001/archgenpsychiatry.2010.71 20603452

[11] De Bellis MD Woolley DP Hooper SR Neuropsychological Findings in Pediatric Maltreatment: Relationship of PTSD, Dissociative Symptoms, and Abuse/ Neglect Indices to Neurocognitive Outcomes. Child Maltreat (2013) 18:171–83. 10.1177/1077559513497420 PMC3769175 23886642

[12] Su Y D'Arcy C Yuan S Meng X How does childhood maltreatment influence ensuing cognitive functioning among people with the exposure of childhood maltreatment? A systematic review of prospective cohort studies. J Affect Disord (2019) 252:278–93. 10.1016/j.jad.2019.04.026 30991256

[13] Malarbi S Abu-Rayya HM Muscara F Stargatt R Neuropsychological functioning of childhood trauma and post-traumatic stress disorder: A meta-analysis. Neurosci Biobehav Rev (2017) 72:68–86. 10.1016/j.neubiorev.2016.11.004 27851897

[14] Aas M Steen NE Agartz I Aminoff SR Lorentzen S Sundet K Is cognitive impairment following early life stress in severe mental disorders based on specific or general cognitive functioning? Psychiatry Res (2012) 198:495–500. 10.1016/j.psychres.2011.12.045 22472845

[15] O'Doherty DCM Chitty KM Saddiqui S Bennett MR Lagopoulos J A systematic review and meta-analysis of magnetic resonance imaging measurement of structural volumes in posttraumatic stress disorder. Psychiatry Res Neuroimaging (2015) 232:1–33. 10.1016/j.pscychresns.2015.01.002 25735885

[16] Woon FL Sood S Hedges DW Hippocampal volume deficits associated with exposure to psychological trauma and posttraumatic stress disorder in adults: a meta-analysis. Prog Neuropsychopharmacol Biol Psychiatry (2010) 34:1181–8. 10.1016/j.pnpbp.2010.06.016 20600466

[17] Teicher MH Anderson CM Polcari A Childhood maltreatment is associated with reduced volume in the hippocampal subfields CA3, dentate gyrus, and subiculum. Proc Natl Acad Sci U S A (2012) 109:E563–72. 10.1073/pnas.1115396109 PMC3295326 22331913

[18] Teicher MH Samson JA Anderson CM Ohashi K The effects of childhood maltreatment on brain structure, function and connectivity. Nat Rev Neurosci (2016) 17:652–66. 10.1038/nrn.2016.111 27640984

[19] Nagae N Hirohata S Shimura Y Yamada S Foa EB Nedate K Japanese version of Posttraumatic Diagnostic Scale: The reliability and validity among Japanese student population. Jpn J Trauma Stress (2007) 5:51–6. 10.1037/t66692-000

[20] Foa EB PDS: Posttraumatic Stress Diagnostic Scale: Manual. Minneapolis: Pearson (1995).

[21] Sheehan DV Lecrubier Y Sheehan KH Amorim P Janavs J Weiller E The Mini-International Neuropsychiatric Interview (M.I.N.I.): the development and validation of a structured diagnostic psychiatric interview for DSM-IV and ICD-10. J Clin Psychiatry (1998) 59(Suppl 20):22–33. 10.4088/JCP.09m05305whi 9881538

[22] Blake DD Weathers FW Nagy LM Kaloupek DG Gusman FD Charney DS The development of a clinician-administered PTSD scale. J Trauma Stress (1995) 8:75–90. 10.1002/jts.2490080106 7712061

[23] Itoh M Ujiie Y Nagae N Niwa M Kamo T Lin M The Japanese version of the Posttraumatic Diagnostic Scale: validity in participants with and without traumatic experiences. Asian J Psychiatr (2017) 25:1–5. 10.1016/j.ajp.2016.09.006 28262126

[24] Asukai N Kato H Kawamura N Kim Y Yamamoto K Kishimoto J Reliability and validity of the Japanese-language version of the impact of event scale-revised (IES-R-J): four studies of different traumatic events. J Nerv Ment Dis (2002) 190:175–82. 10.1097/00005053-200203000-00006 11923652

[25] Weiss DS Marmar CR The Impact of Event Scale-Revised. In: Wilson JP Keanem TM, editors. Assessing Psychological Trauma and PTSD: A Practitioner"s Handbook. New York: Guilford Press (2004). p. 399–411.

[26] Spielberger C Gorsuch R Lushene R Manual for the state- trait anxiety inventory. Palo Alto, CA: Consulting Psychologists Press (1970).

[27] Nakazato K Mizuguchi K Development of a Japanese version of new anxiety scale State–Trait Anxiety Inventory (STAI). Jpn J Psychosom Med (1982) 22:108–12. 10.15064/jjpm.22.2_107

[28] Beck AT Steer RA Brown GK Beck Depression Inventory manual-II. San Antonio, TX: Psychological Corporation (1996).

[29] Kojima M Furukawa TA Takahashi H Kawai M Nagaya T Tokudome S Cross-cultural validation of the Beck Depression Inventory-II in Japan. Psychiatry Res (2002) 110:291–9. 10.1016/S0165-1781(02)00106-3 12127479

[30] Bernstein DP Stein JA Newcomb MD Walker E Pogge D Ahluvalia T Development and validation of a brief screening version of the Childhood Trauma Questionnaire. Child Abuse Negl (2003) 27:169–90. 10.1016/S0145-2134(02)00541-0 12615092

[31] Bernstein DP Fink L Childhood Trauma Questionnaire: A retrospective self-report manual. San Antonio, TX: The Psychological Corporation (1998).

[32] Scher CD Stein MB Asmundson GJ McCreary DR Forde DR The childhood trauma questionnaire in a community sample: psychometric properties and normative data. J Trauma Stress (2001) 14:843–57. 10.1023/A:1013058625719 11776429

[33] Gerdner A Allgulander C Psychometric properties of the Swedish version of the Childhood Trauma Questionnaire-Short Form (CTQ-SF). Nord J Psychiatry (2009) 63:160–70. 10.1080/08039480802514366 19021077

[34] Matsui M Kasai Y Nagasaki M Reliability and validity of the Japanese version of the Repeatable Battery for the Assessment of Neuropsychological Status [in Japanese]. Toyama Med J (2010) 21:31–6. 10.15099/00016036

[35] Randolph C Tierney MC Mohr E Chase TN The Repeatable Battery for the Assessment of Neuropsychological Status (RBANS): preliminary clinical validity. J Clin Exp Neuropsychol (1998) 20:310–9. 10.1076/jcen.20.3.310.823 9845158

[36] Duff K Beglinger LJ Schoenberg MR Patton DE Mold J Scott JG Test-retest stability and practice effects of the RBANS in a community dwelling elderly sample. J Clin Exp Neuropsychol (2005) 27:565–75. 10.1080/13803390490918363 16019633

[37] McKay C Casey JE Wertheimer J Fichtenberg NL Reliability and validity of the RBANS in a traumatic brain injured sample. Arch Clin Neuropsychol (2007) 22:91–8. 10.1016/j.acn.2006.11.003 17141467

[38] Weber B RBANS has reasonable test-retest reliability in schizophrenia. Evid Based Ment Health (2003) 6:22. 10.1136/ebmh.6.1.22 12588828

[39] Dauvermann MR Donohoe G The role of childhood trauma in cognitive performance in schizophrenia and bipolar disorder - A systematic review. Schizophr Res Cogn (2019) 16:1–11. 10.1016/j.scog.2018.11.001 30581765 PMC6293032

[40] Bücker J Kozicky J Torres IJ Kauer-Sant'anna M Silveira LE Bond DJ The impact of childhood trauma on cognitive functioning in patients recently recovered from a first manic episode: data from the Systematic Treatment Optimization Program for Early Mania (STOP-EM). J Affect Disord (2013) 148:424–30. 10.1016/j.jad.2012.11.022 23246364

[41] Masson M East-Richard C Cellard C A meta-analysis on the impact of psychiatric disorders and maltreatment on cognition. Neuropsychology (2016) 30:143–56. 10.1037/neu0000228 26192540

[42] Lereya ST Samara M Wolke D Parenting behavior and the risk of becoming a victim and a bully/victim: a meta-analysis study. Child Abuse Negl (2013) 37:1091–108. 10.1016/j.chiabu.2013.03.001 23623619

[43] Bellis MA Hughes K Ford K Hardcastle KA Sharp CA Wood S Adverse childhood experiences and sources of childhood resilience: a retrospective study of their combined relationships with child health and educational attendance. BMC Public Health (2018) 18:792. 10.1186/s12889-018-5699-8 29940920 PMC6020215

[44] Jonas S Bebbington P McManus S Meltzer H Jenkins R Kuipers E Sexual abuse and psychiatric disorder in England: results from the 2007 Adult Psychiatric Morbidity Survey. Psychol Med (2011) 41:709–19. 10.1017/S003329171000111X 20534178

[45] Hailes HP Yu R Danese A Fazel S Long-term outcomes of childhood sexual abuse: an umbrella review. Lancet Psychiatry (2019) 6:830–9. 10.1016/S2215-0366(19)30286-X PMC7015702 31519507

[46] Vonderlin R Kleindienst N Alpers GW Bohus M Lyssenko L Schmahl C Dissociation in victims of childhood abuse or neglect: a meta-analytic review. Psychol Med (2018) 48:2467–76. 10.1017/S0033291718000740 29631646

[47] Baumeister D Akhtar R Ciufolini S Pariante CM Mondelli V Childhood trauma and adulthood inflammation: a meta-analysis of peripheral C-reactive protein, interleukin-6 and tumour necrosis factor-α. Mol Psychiatry (2016) 21:642–9. 10.1038/mp.2015.67 PMC4564950 26033244

[48] Bunea IM Szentágotai-Tătar A Miu AC Early-life adversity and cortisol response to social stress: a meta-analysis. Transl Psychiatry (2017) 7:1274. 10.1038/s41398-017-0032-3 29225338 PMC5802499

[49] Tyrka AR Ridout KK Parade SH Childhood adversity and epigenetic regulation of glucocorticoid signaling genes: Associations in children and adults. Dev Psychopathol (2016) 28(4pt2):1319–31. 10.1017/S0954579416000870 PMC5330387 27691985

[50] Lupien SJ McEwen BS Gunnar MR Heim C Effects of stress throughout the lifespan on the brain, behaviour and cognition. Nat Rev Neurosci (2009) 10:434–45. 10.1038/nrn2639 19401723

[51] Spooren A Kolmus K Laureys G Clinckers R De Keyser J Haegeman G Interleukin-6, a mental cytokine. Brain Res Rev (2011) 67:157–83. 10.1016/j.brainresrev.2011.01.002 21238488

[52] Imai R Hori H Itoh M Lin M Niwa M Ino K Inflammatory markers and their possible effects on cognitive function in women with posttraumatic stress disorder. J Psychiatr Res (2018) 102:192–200. 10.1016/j.jpsychires.2018.04.009 29684628

[53] Yehuda R Bierer LM Schmeidler J Aferiat DH Breslau I Dolan S Low cortisol and risk for PTSD in adult offspring of holocaust survivors. Am J Psychiatry (2000) 157:1252–9. 10.1176/appi.ajp.157.8.1252 10910787

[54] Eraly SA Nievergelt CM Maihofer AX Barkauskas DA Biswas N Agorastos A Assessment of plasma C-reactive protein as a biomarker of posttraumatic stress disorder risk. JAMA Psychiatry (2014) 71:423–31. 10.1001/jamapsychiatry.2013.4374 PMC4032578 24576974

[55] Parslow RA Jorm AF Pretrauma and posttrauma neurocognitive functioning and PTSD symptoms in a community sample of young adults. Am J Psychiatry (2007) 164:509–15. 10.1176/ajp.2007.164.3.509 17329477

[56] Nolin P Ethier L Using neuropsychological profiles to classify neglected children with or without physical abuse. Child Abuse Negl (2007) 31:631–43. 10.1016/j.chiabu.2006.12.009 17617456

[57] Ritchie K Jaussent I Stewart R Dupuy AM Courtet P Malafosse A Adverse childhood environment and late-life cognitive functioning. Int J Geriatr Psychiatry (2011) 26:503–10. 10.1002/gps.2553 21445999

[58] Baldwin JR Reuben A Newbury JB Danese A Agreement Between Prospective and Retrospective Measures of Childhood Maltreatment: A Systematic Review and Meta-analysis. JAMA Psychiatry (2019) 76:584–93. 10.1001/jamapsychiatry.2019.0097 PMC6551848 30892562

